# Leucine Supplementation Accelerates Connective Tissue Repair of Injured Tibialis Anterior Muscle

**DOI:** 10.3390/nu6103981

**Published:** 2014-09-29

**Authors:** Marcelo G. Pereira, Meiricris T. Silva, Eduardo O. C. Carlassara, Dawit A. Gonçalves, Paulo A. Abrahamsohn, Isis C. Kettelhut, Anselmo S. Moriscot, Marcelo S. Aoki, Elen H. Miyabara

**Affiliations:** 1 Department of Anatomy, Institute of Biomedical Sciences, University of Sao Paulo, Prof. Lineu Prestes Av. 2415, Sao Paulo, SP 05508-000, Brazil; E-Mails: pereiramg@gmail.com (M.G.P.); me_tomaz@hotmail.com (M.T.S.); eduardo.carlassara@usp.br (E.O.C.C.); moriscot@usp.br (A.S.M.); 2 Department of Physiology and Biochemistry/Immunology, Ribeirao Preto Medical School, University of Sao Paulo, Bandeirantes Av. 3900, Ribeirao Preto, SP 14049-900, Brazil; E-Mails: dawitapg@yahoo.com.br (D.A.G.); idckette@fmrp.usp.br (I.C.K.); 3 Department of Cell and Developmental Biology, Institute of Biomedical Sciences, University of Sao Paulo, Prof. Lineu Prestes Av. 1524, Sao Paulo, SP 05508-000, Brazil; E-Mail: pauloabrahamsohn@gmail.com; 4 School of Arts, Sciences and Humanities, University of Sao Paulo, Arlindo Bettio Av. 1000, Sao Paulo, SP 03828-000, Brazil; E-Mail: aoki.ms@usp.br

**Keywords:** skeletal muscle injury, repair, leucine supplementation, connective tissue, transforming growth factor-β receptor type I, Smad2/3

## Abstract

This study investigated the effect of leucine supplementation on the skeletal muscle regenerative process, focusing on the remodeling of connective tissue of the fast twitch muscle tibialis anterior (TA). Young male Wistar rats were supplemented with leucine (1.35 g/kg per day); then, TA muscles from the left hind limb were cryolesioned and examined after 10 days. Although leucine supplementation induced increased protein synthesis, it was not sufficient to promote an increase in the cross-sectional area (CSA) of regenerating myofibers (*p* > 0.05) from TA muscles. However, leucine supplementation reduced the amount of collagen and the activation of phosphorylated transforming growth factor-β receptor type I (TβR-I) and Smad2/3 in regenerating muscles (*p* < 0.05). Leucine also reduced neonatal myosin heavy chain (MyHC-*n*) (*p* < 0.05), increased adult MyHC-II expression (*p* < 0.05) and prevented the decrease in maximum tetanic strength in regenerating TA muscles (*p* < 0.05). Our results suggest that leucine supplementation accelerates connective tissue repair and consequent function of regenerating TA through the attenuation of TβR-I and Smad2/3 activation. Therefore, future studies are warranted to investigate leucine supplementation as a nutritional strategy to prevent or attenuate muscle fibrosis in patients with several muscle diseases.

## 1. Introduction

Adult skeletal myofibers have a remarkable capacity of regeneration after muscle damage [1]. The muscle regenerative process includes migration of immune cells, such as neutrophils and macrophages, to the site of injury, the proliferation and differentiation of satellite cells and the remodelling of connective tissue and angiogenesis, resulting in the functional recovery of damaged muscles [2,3]. Recovery of the skeletal muscle mass is a crucial event during skeletal muscle regeneration [4,5]. Intracellular signaling pathways that control protein turnover are strongly modulated by this process [6,7]. As previously established by us and others, the phosphoinositide 3-kinase (PI3K)/Akt/mechanistic target of the rapamycin pathway and the ubiquitin proteasome system play significant roles in skeletal muscle mass recovery after injury [5,6,7,8,9,10]. The growth of regenerating myofibers is dependent on various processes, such as satellite cell proliferation, myotube formation, angiogenesis and the re-establishment of neuromuscular and connective tissue. Regenerated myofibers can be recognized by the presence of centrally located nuclei, which are a hallmark of muscle regeneration [11].

The re-establishment of the skeletal muscle extracellular matrix (ECM) is a crucial event during muscle regeneration. Deficits in ECM modulation in damaged muscles, with the consequent accumulation of connective tissue, generically termed fibrosis, has important clinical manifestations, such as the impairment of muscle force transmission, infiltration of inflammatory cells and disruption of the basal lamina [12]. Additionally, it is well known that the transforming growth factor β (TGF-β)/Smad signaling pathway is a strong stimulator of fibrosis [13].

Although the process of skeletal muscle regeneration has been investigated in detail for decades at the morphological and cellular level, studies on the strategies to improve this process are still on going. Therefore, the use of potential agents capable of modulating the ECM in order to maximize the recovery of muscle tissue after injury may be a suitable strategy. A good candidate is leucine, an essential amino acid, known not only as a component of proteins, but also as a physiopharmacological agent that promotes important anti-catabolic actions, such as attenuation of skeletal muscle catabolism during immobilization [14], facilitation of the healing process [15] and improvement in skeletal muscle protein turnover in aged individuals [16,17]. Recently, it has been demonstrated that the leucine-rich, internal region of the proteoglycan decorin interacts with the low-density lipoprotein receptor-related protein-1, modulating TGF-β-dependent signaling and, consequently, inhibiting the TGF-β-dependent fibrotic response in skeletal muscles [18]. In addition, leucine has reportedly improved the overall morphology of regenerating muscles [19], and more recently, we have showed that this effect involves reduction of FOXO3a activation and ubiquitinated protein accumulation [10]. However, the putative role of leucine in modulating connective tissue repair during muscle regeneration is unknown. Therefore, the aim of the present study was to investigate the effect of leucine supplementation on the connective tissue recovery of regenerating tibialis anterior (TA) muscles in rats.

## 2. Experimental Section

The protocols used in this study were in agreement with the ethical principles in animal research followed by the Brazilian College of Animal Experimentation and were approved by the Ethics Committee in Animal Research of the Institute of Biomedical Sciences at the University of Sao Paulo (Protocol NO. 87/2011).

### 2.1. Animals and Leucine Supplementation

Two-month old male Wistar rats (*n* = 22) weighing 292.1 ± 25.2 g were housed in standard plastic cages in an animal facility with controlled environmental conditions (24 °C; 12/12-h light/dark cycle) and reared on standard food and water *ad libitum*. Beginning 3 days prior to muscle cryolesion and continuing until the end of experimental period (Day 10 post-cryolesion), l-leucine (Ajinomoto, Tokyo, Japan) was administered once a day by oral gavage at a dose of 1.35 g/kg body mass [20,21,22]. A control group received saline only. Leucine was dissolved in distilled water, and each animal was gavaged with a 5-mL volume of distilled water [20]. In our previous experiments, muscles from saline-gavaged rats did not show morphological changes compared to those from intact animals (data not shown).

Due to methodological requirements of the *ex vivo* experiments to measure the rate of protein synthesis, leucine supplementation was initiated in three-week old male Wistar rats (*n* = 7) weighing 56.1 ± 4.0 g, and the muscle submitted to the cryolesion was the extensor digitorum longus (EDL). These adaptations were performed in order to allow an adequate diffusion of metabolites and oxygen in the entire muscle. Therefore, previous experiments were carefully executed to confirm that in these younger rats, the cryolesioned EDL muscles supplemented with leucine had similar morphological effects when compared to cryolesioned TA muscles supplemented with leucine from 2-month old rats (*i.e*., a reduction of inflammatory cell infiltration and unchanged myofiber cross-sectional area when compared to those from the cryolesion only (Cryo) group; data not shown).

### 2.2. Experimental Design

In order to investigate the effects of leucine supplementation on muscle regeneration, rats received leucine supplementation or not and were submitted to cryolesion of TA muscles of the left hind limb. The contralateral muscles (right hind limb) were used as the intact control [4]. TA muscles were randomly divided into four groups: control (C, right hind limb from untreated animal; *n* = 6); leucine supplementation only (Leu, right hind limb from supplemented animal; *n* = 6); cryolesion only (Cryo, left hind limb from untreated animal; *n* = 6) and leucine supplementation combined with cryolesion (Cryo + Leu, left hind limb from supplemented animal; *n* = 6). To assess the effects of leucine supplementation on muscle strength, additional animals from each group (*n* = 5) were used [4,5]. Prior to cryolesion, animals were anaesthetized with ketamine and xylazine (5 mg/100 g body mass, i.p.), and all efforts were made to minimize suffering. The left TA muscle was surgically exposed until the total surface of the muscle was completely visible. The cryolesion consisted of one freeze-thaw cycle of the muscles *in situ*. In order to freeze the entire muscle surface, an iron rod was pre-cooled in liquid nitrogen, after which the flat end (0.4 × 1.0 cm) was brought into contact with the superior surface of the muscle for 10 s, three times. This procedure was performed in all groups to avoid varying amounts of damage to the TA muscles from each group. After the muscles were thawed, the wounds were closed with 3-0 silk sutures. For several minutes thereafter, animals were held on a warming plate at 37 °C to avoid hypothermia. The contralateral TA muscles (right hind limb) were used as intact controls [4,5].

On Day 10 post-cryolesion, animals received the last dose of leucine, were euthanized one hour later, and their left and right TA muscles were removed and weighed. Similar procedures were performed in EDL muscles.

### 2.3. Morphometric and Quantitative Analyses

Each muscle was individually frozen in melting isopentane, stored in liquid nitrogen and had its middle belly cut into 10-µm cross-sections on a cryostat (CM3050; Leica, Nussloch, Germany). Unfixed muscle cross-sections were stained with a solution of aqueous toluidine blue and borax (1% w/v for both) to reveal the overall morphology and analyzed under a light microscope (PCM 2000; Nikon, Melville, New York, NY, USA). Quantification of the connective tissue area density was performed by the Sirius red method and analyzed under light and polarized microscopes (PCM 2000; Nikon, Melville, New York, NY, USA), as previously reported [23,24]. Morphometric and quantitative analyses were conducted with a digitizing unit linked to computer software (Image-Pro Plus; Media Cybernetics, Rockville, MD, USA). To determine myofiber cross-sectional area (CSA; µm^2^), a total of approximately 500 myofibers per muscle was measured. In the non-injured groups, CSA measurements were obtained from toluidine blue-stained muscle fields that were randomly taken. In the cryolesion groups, CSA measurements were obtained only from toluidine blue-stained muscle fields obtained from the regenerating area, *i.e.*, the area characterized by the exclusive presence of myofibers with centralized nuclei [5]. Five entire muscle cross-sections per animal were used for quantification of inflammatory area, which was characterized by the presence of clear areas between myofibers containing inflammatory cell infiltration observed in muscles stained with toluidine blue [5]. In addition, a planimetry system was used for the analysis of the intramuscular connective tissue density by scoring the points containing 450 line intersections per field. The coincident points in the endomysium and perimysium in three areas per section and five muscle cross-sections per animal corresponded to a total of 6750 points per animal. The area density, which is the relative area of connective tissue, was calculated by dividing the sum of the number of coincident points in straight-line intersections in the connective tissues (endomysium and perimysium) by the total number of points. The connective tissue area density and the inflammation area were expressed as a percentage of whole muscle cross-section [25].

### 2.4. Immunostaining

Unfixed muscle cross-sections were immunostained against MyHC-*n* antibody by using the Vectastain Elite ABC Kit (Vector Laboratories, Burlingame, CA, USA), according to the manufacturer’s recommendations. The sections were then incubated with the 3,3ʹ-diaminobenzidine substrate kit for peroxidase (Vector Laboratories) and counterstained with hematoxylin. The primary antibody used was a monoclonal mouse anti-neonatal myosin heavy chain (MyHC-*n*; 1:20; RNMy2/9D2, Novocastra, Newcastle upon Tyne, UK). The following secondary antibody used was a mouse IgG conjugated to horseradish peroxidase (1:20; Vectastain Elite ABC Kit, Vector Laboratories). The MyHC-*n* positive regenerating cells were counted in the entire muscle cross-section, and the total area of the section was measured using the software Image-Pro Plus; thus, the data were expressed as the percentage of whole-muscle CSA [5].

Muscle cross-sections to be used for immunodetection of TβRI and Smad2/3 were fixed with 4% paraformaldehyde in 0.2 M phosphate buffer (PB) for 10 min at room temperature, blocked with 0.1 glycine in phosphate-buffered saline (PBS) for 5 min and permeabilized in 0.2% Triton X-100/PBS for 10 min. The slides were incubated overnight in a moisture chamber at 4 °C with a solution containing the primary antibodies together with 3% normal goat serum and 0.3% Triton X-100/0.1 M PB. After the slides had been washed (three 10-min washes with 0.1 M PB), a solution containing the respective secondary antibodies and 0.3% Triton X-100/0.1 M PB was added, and the slides were maintained in this solution for 2 h in a dark room. The slides were again washed in 0.1 M PB (three 10-min washes), after which they were mounted with Vectashield mounting medium containing 4ʹ,6-diamidino-2-phenylindole (Vector Laboratories) and cover-slipped. The primary antibodies used were: rabbit monoclonal anti-Smad2/3 (1:200; Cell Signaling Technology, Massachusetts, MA, USA); rabbit polyclonal anti-TβRI phospho S165 (1:250; AbCam, Cambridge, MA, USA). The following secondary antibody was used: Rhodamine anti-rabbit (1:200; AbCam, Cambridge, MA, USA).

Tissue cross-sections positive for TβR-I and the number of nuclei positive for Smad2/3 were assessed from four fields, randomly taken from the regenerating area at 10 days post-cryolesion; and the total area of the section was measured using the software, Image-Pro Plus. The percentage of TβR-I-positive areas and the number of Smad2/3-positive nuclei were calculated considering the area of four fields (mm^2^). Since the expression of phosphorylated TβR-I and Smad2/3 positive nuclei were absent in the C and Leu groups, the quantification of them in these groups was not performed.

The stained sections were analyzed in a fluorescence microscope (Observer D1, Zeiss, Jena, Germany). Figures were mounted using Adobe Photoshop v7.0, with image manipulation being restricted to overall threshold and brightness adjustments.

### 2.5. Hydroxyproline Content

Hydroxyproline content in muscles was quantified [26] by colorimetric assay using a commercial kit (Hydroxyproline Assay Kit, BioVision Inc., Milpitas, CA, USA), according to manufacturer’s instructions.

### 2.6. Ex Vivo Protein Synthesis Measurement

According to Gonçalves and coworkers [27], EDL muscles were rapidly dissected, weighed and maintained at approximately their resting length by pinning the tendons on inert plastic supports. Tissues were incubated at 37 °C in Krebs-Ringer bicarbonate buffer (pH 7.4) equilibrated with 95% oxygen and 5% carbon dioxide, containing glucose (5 mmol/L) and all amino acids at concentrations similar to those of rat plasma [28]. After a 1-h equilibration period, l-[U-^14^C] tyrosine (0.05 µCi/mL) was added to the replacement medium, and the muscles were incubated on that for 2 h. Subsequently, the estimation of the specific activity of acid-soluble tyrosine (intracellular tyrosine pool) in each muscle was performed by measuring the radioactivity and the concentration of tyrosine in this pool, which was determined by the method of Waalkes and Udenfriend [29]. After measurement of the radioactivity incorporated into total protein of the same muscle, the rate of synthesis was calculated using the specific activity of the intracellular pool of tyrosine of each muscle, assuming that there was no recycling of the label during the incubation time [30,31].

### 2.7. Western Blotting Analysis

Muscle samples were homogenized in an extraction solubilization buffer, composed of 90 mM KCl, 10 mM 4-2-hydroxyethyl-1-piperazineethanesulfonic acid, 3 mM MgCl^2+^, 5 mM ethylenediaminetetraacetic acid (EDTA), 1% glycerol, 1 mM dithiothreitol, 0.04% sodium dodecyl sulfate, proteinase and phosphatase inhibitor cocktail (1:100; Sigma-Aldrich, St. Louis, MO, USA). Homogenates were centrifuged at 12,000× *g* for 10 min at 4 °C, the supernatant was collected, and the protein was quantified by the Bradford assay (Bio-Rad, Hercules, CA, USA) with bovine serum albumin as the standard [32]. Equal amounts of protein (50 µg) were separated on 6% sodium dodecyl sulfate-polyacrylamide gels, electrophoresed and transferred to a nitrocellulose membrane (Bio-Rad, USA). The membranes were stained with Ponceau S to determine the protein content and rinsed with Tris-buffered saline/Tween solution (0.5 M NaCl; 50 mM Tris-HCl, pH 7.4; and 0.1% Tween 20). Membranes were incubated overnight at 4 °C with primary antibodies. After a 30-min wash in Tris-buffered saline/Tween solution, membranes were incubated with secondary antibodies for 1 h at room temperature. The membranes were again washed for 30 min in Tris-buffered saline/Tween solution. Detection of the labelled proteins was achieved using the enhanced chemiluminescence system (ECL; Amersham, Pittsburgh, PA, USA) and autoradiography. Densitometry analysis was performed by using ImageJ software (Scion Corp., National Institutes of Health, Bethesda, MD, USA). Experiments were performed on four separate samples from each group.

The primary antibodies used for western blotting were mouse polyclonal antibodies raised against fast myosin heavy chain (MyHC-II) (1:500; AbCam, Cambridge, MA, USA), rabbit polyclonal antibodies raised against phosphor-p70^S6K^ at the Ser^371^ and Thr^389^ residues, 4E-BP1 and eIF4E (1:1,000; Cell Signaling Technology, USA). In addition, we used a rabbit polyclonal antibody raised against ubiquitin (1:1,500; Boston Biochem, Cambridge, MA, USA). Targeted bands were normalized to glyceraldehyde-3-phosphate dehydrogenase (GAPDH, 1:1,000; Cell Signaling Technology, USA). The secondary antibodies used were peroxidase-conjugated goat anti-mouse IgG and goat anti-rabbit IgG (AffiniPure, 1:10,000; Jackson ImmunoResearch Laboratories Inc., West Grove, PA, USA).

### 2.8. Muscle Function Experiments in Vivo

Skeletal muscle function analyses were performed as previously described [10]. Animals were anaesthetized with tribromoethanol (20 mg/100 g of body weight, i.p.) The sciatic nerve was exposed through a lateral incision on the thigh, and an electrode was connected. Sciatic nerve innervations to the TA muscle were carefully isolated from those originating from other nerves. In order to immobilize the limb, animals were placed on an acrylic platform with a metal bar crossing the knee to fix the limb. The hind foot was fastened to another metal bar, and the TA tendon was connected to a force transducer coupled to a computer that was used to collect and analyze data related to the strength generated by muscle contraction. Muscle twitch and maximum tetanic strength were recorded using a data acquisition system (Biopac Systems, Goleta, CA, USA), whereas muscle strength was analyzed with AcqKnowledge software, version 3.9.1.6 (Biopac Systems, USA). Rats were submitted to external warming in order to maintain their core temperature throughout the procedure.

At the initiation of the experiment, each muscle was set to the optimum length (L_0_, defined as the length resulting in maximum twitch strength), as well as a 2-min rest period between stimuli [33]. To achieve the maximal plateau strength with minimal frequency, we used 200-Hz stimuli for TA for measuring the maximum tetanic strength and 200 Hz to evaluate fatigue.

Isolated twitches (0.2 Hz) were generated over a 2-min period, followed by a pre-fatigue maximum tetanic contraction (induced for 2 s) in each muscle (at 200 Hz) [34]. We then performed a fatigue protocol, which consisted of ten 2-s stimulations (at 200 Hz tetanus), each followed by a 4-s rest. At the end of the fatigue protocol, a 2-min rest-period was given to the muscle by stimulating it at 0.2 Hz, followed by a post-fatigue maximum tetanic contraction. Among these groups, we observed no differences in terms of twitch parameters, such as the time-to-peak and half-relaxation time (data not shown). Development of muscle fatigue was measured at four time points (first, fourth, seventh and tenth contractions). Maximum tetanic strength and fatigue were expressed in millinewtons.

### 2.9. Statistical Analysis

Data are presented as the mean and standard deviation. We used mixed models for repeated measurements in order to evaluate the effects of cryolesion and leucine supplementation on maximum tetanic strength and on the development of muscle fatigue. Analysis of variance (the general linear model) was used in order to evaluate the effects of cryolesion and leucine supplementation on the area density of connective tissue, hydroxyproline content, protein expression analysis, rate of protein synthesis, quantification of TGF-β receptor positive area and Smad2/3 positive nuclei. A Kolmogorov-Smirnov test was performed to compare the frequency distribution of myofiber CSA’s of the groups. The Student’s *t*-test was used in order to evaluate the effects that leucine supplementation has on inflammatory area and MyHC-*n* expression. Whenever a significant *F*-value was obtained, Tukey’s *post hoc* test was performed for multiple comparison purposes (SAS 9.2 software; SAS Institute Inc., Cary, NC, USA). Values of *p* < 0.05 were considered statistically significant.

## 3. Results

### 3.1. Muscle Morphological Features

**Figure 1:**
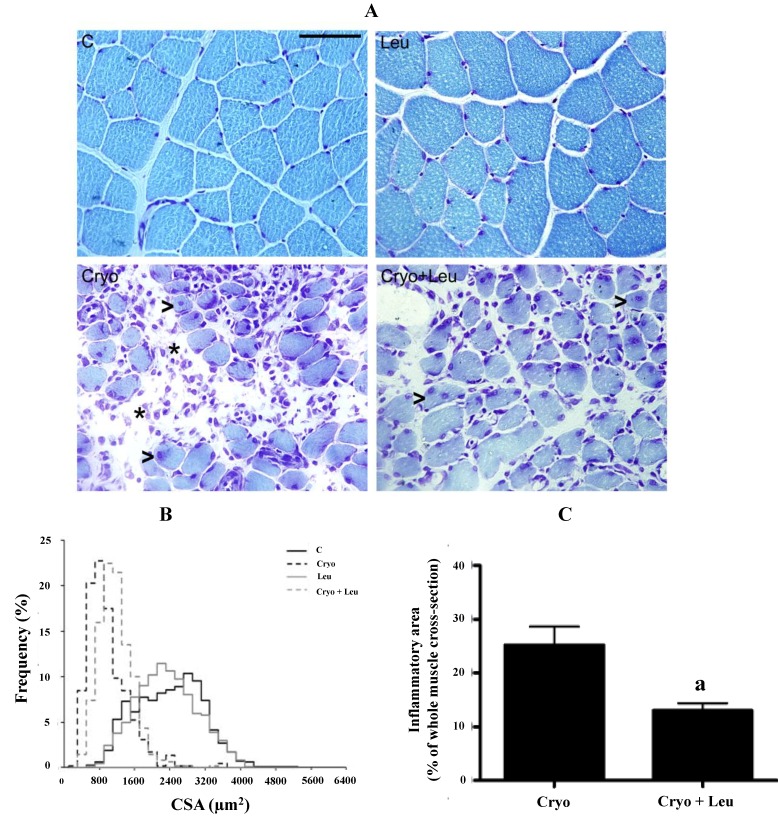
Histological features of tibialis anterior (TA) muscle cross-sections (**A**). The frequency distribution of myofiber CSA (**B**). Inflammatory area of TA muscles at 10 days post-cryolesion (**C**). C, control muscles; Leu, muscles after 13 days of leucine supplementation; Cryo, muscles analyzed on Day 10 post-cryolesion; Cryo + Leu, leucine supplemented group analyzed on Day 10 post-cryolesion. Note that the intact control muscles (**C**) and the leucine supplemented-only muscles (Leu) have a normal morphology. On Day 10 post-cryolesion, the Cryo and Cryo + Leu group muscles present regenerating myofiber with centralized nuclei (arrowheads in Cryo and Cryo + Leu groups in (**A**)), and there is less inflammatory infiltration in the Cryo + Leu group (asterisks in Cryo and Cryo + Leu groups in (**A**)). Toluidine blue staining. Scale bar = 50 µm.

Histological cross-sections of TA muscles from animals examined on Day 10 post-cryolesion were stained with toluidine blue and analyzed microscopically. We noted that the normal tissue structure of intact muscles exhibited myofibers with peripheral nuclei and polygonal shape (Figure 1A). TA muscle from the Leu group presented 12% of myofibers larger than 3200 µm^2^, while TA from the C group had 14% of myofibers larger than 3200 µm^2^ (*p* > 0.05, Figure 1B).

On Day 10 post-cryolesion, muscles showed myofibers with various stages of regeneration, as indicated by the presence of centrally nucleated myofibers of various sizes and by the infiltration of inflammatory cells (Figure 1A). TA muscles from the Cryo group had 10% of myofibers larger than 1600 µm^2^, whereas muscles from Cryo + Leu group presented 9% of myofibers larger than 1600 µm^2^ (*p* > 0.05, Figure 1B).

In addition, there was a decrease in inflammatory area on TA muscles from the Cryo + Leu group when compared to those from the Cryo group (48%; *p* < 0.05, Figure 1C).

### 3.2. Skeletal Muscle Function Measurements

**Figure 2:**
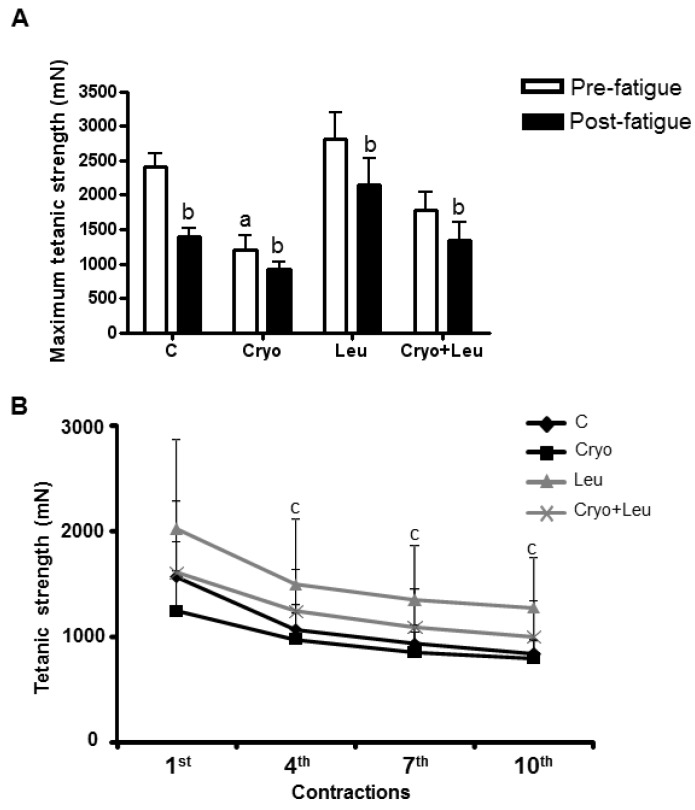
Maximum tetanic strength at pre-fatigue and post-fatigue protocol of TA muscles (**A**) analyzed on Day 10 post-cryolesion. ^a^ *p* *<* 0.05 *vs.* C and Leu; ^b^ *p* *<* 0.05 *vs.* pre-fatigue tetanic contraction from the same group. Development of muscle fatigue analyzed at four time points (first, fourth, seventh and tenth contractions) on TA muscles (**B**) on Day 10 post-cryolesion. ^c^ *p* *<* 0.05 *vs.* first contraction in all groups. C, intact control muscles; Cryo, muscles analyzed on Day 10 post-cryolesion; Leu, muscles after 13 days of leucine supplementation; Cryo + Leu, leucine supplemented group analyzed on Day 10 post-cryolesion. Muscle strength is expressed in millinewtons (mN). Data are presented as the mean ± SD.

There was a decrease of post-fatigue maximum tetanic contraction in TA muscles from both the C (43%) and Leu (25%) groups when compared to their pre-fatigue maximum tetanic contractions (*p* < 0.05, Figure 2A). In the Cryo group, the pre-fatigue maximum tetanic contraction was 50% lower when compared to that from C group (*p* < 0.05, Figure 2A), whereas the post-fatigue maximum tetanic contraction decreased 24% when compared to the pre-fatigue (*p* < 0.05, Figure 2A). No difference was observed in pre-fatigue maximum tetanic contraction in the TA muscle from the Cryo + Leu group when compared to that from the C group, whereas there was a decrease in the post-fatigue maximum tetanic contraction when compared to the pre-fatigue in the Cryo + Leu group (25%, *p* < 0.05, Figure 2A).

The strength of TA muscles was measured at ten time points during the fatigue protocol, and four of these time points were used to describe the fatigue development (first, fourth, seventh and tenth contractions). A significant loss of TA strength from C group was observed (30%), beginning at the fourth contraction (Figure 2B), which was maintained up to the tenth contraction (50%, *p* *<* 0.05, Figure 2B). In the Leu groups, strength development was decreased until it reached 30% at the tenth contraction (*p* *<* 0.05, Figure 2B). In the Cryo group, the first contraction did not significantly decrease (20%) when compared to those from C groups (Figure 2B); however, strength decreased (35%) at the seventh and tenth contractions, in comparison with the first (*p* *<* 0.05, Figure 2B). In the Cryo + Leu group, no difference was observed in the first contraction, compared to that from C group. On the other hand, at the fourth, seventh and tenth contractions, strength production decreased in the Cryo + Leu group muscle up to 35% in comparison with the first contraction (*p* *<* 0.05, Figure 2B).

### 3.3. Expression of MyHC-n and MyHC-II

**Figure 3:**
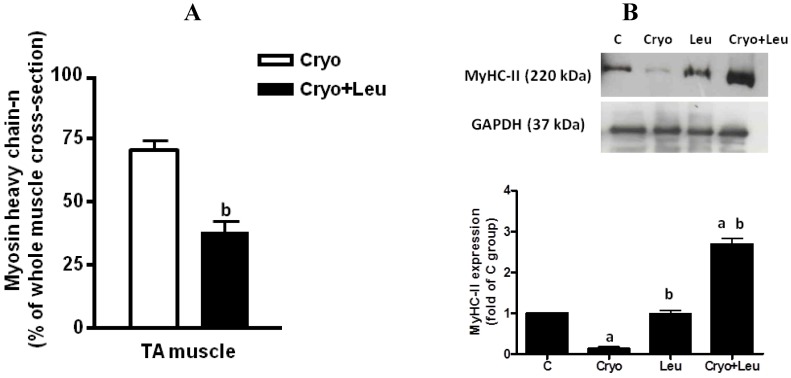
Number of MyHC-*n*-positive regenerating myofibers in TA muscles analyzed on Day 10 post-cryolesion (percentage of whole muscle cross-sectional area; (**A**)). Representative western blots and densitometry analyses of MyHC-II from TA muscles at 10 days post-cryolesion (**B**). Muscle groups are identified at the top. C, control muscles; Cryo, muscles analyzed on Day 10 post-cryolesion; Leu, muscles after 13 days of leucine supplementation; Cryo + Leu, leucine supplemented group analyzed on Day 10 post-cryolesion. GAPDH was used as the standard. Data are expressed as the fold of MyHC-II expression detected in the C group. Molecular weight is presented in kDa. ^a^ *p* < 0.05 *vs.* C and Leu; ^b^ *p* < 0.05 *vs.* Cryo. Data are presented as the mean ± SD.

Immunostaining against MyHC-*n* was performed in order to detect the presence of differentiating myofibers during regeneration. On Day 10 post-cryolesion, TA muscle from the Cryo + Leu group had fewer MyHC-*n* positive regenerating myofibers than did that from the Cryo group (46%, *p* < 0.05, Figure 3A).

In addition, the content of adult MHC-II, the predominant isoform in TA muscle, was also analyzed. No change was observed in the content of MyHC-II in TA muscle between the C and Leu groups (Figure 3B). On Day 10 post-cryolesion, TA muscles presented a decrease in the MyHC-II content when compared to that from the C group (87%, *p* < 0.05, Figure 3B). In addition, there was an increase in the content of MyHC-II on TA muscle from the Cryo + Leu group when compared to those from the C and Cryo groups (*p* < 0.05, 168% and 1900%, respectively; Figure 3B).

### 3.4. Ex Vivo Protein Synthesis, Expression of Elements within the PI3K/Akt/mTOR Pathway and Accumulation of Ubiquitinated Proteins

**Figure 4:**
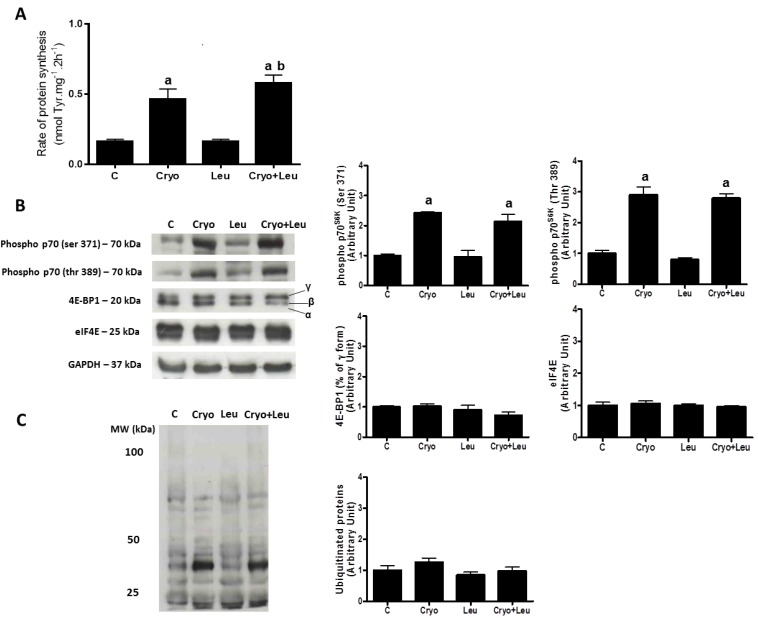
The rate of protein synthesis in extensor digitorum longus (EDL) muscles analyzed on post-cryolesion Day 10 (nmol Tyr·mg^−1^·2 h^−1^; (**A**)). Representative western blots and densitometry analyzes of elements within the PI3K/Akt/mTOR pathway (**B**) and ubiquitinated proteins accumulation (**C**) from TA muscles on post-cryolesion Day 10. Muscle groups are identified at the top. C, control muscles; Cryo, muscles analyzed on post-cryolesion Day 10; Leu, muscles after 13 days of leucine supplementation; Cryo + Leu, leucine supplemented group analyzed on post-cryolesion Day 10. GAPDH was used as the standard. Western blot data are expressed as the fold of protein expression detected in C group. Molecular weight is presented in kDa. ^a^ *p* < 0.05 *vs.* C and Leu; ^b^ *p* < 0.05 *vs.* Cryo. Data are presented as the mean ± SD.

Muscles from the leucine supplemented-only group (Leu) had no changes in the rate of protein synthesis when compared to those from the C group (Figure 4A). However, the muscles from the Cryo group presented an increase of 290% in the rate of protein synthesis when compared to that from the C group, whereas the muscles from the Cryo + Leu group had an increase of 25% in this rate when compared to that from the Cryo group (*p* *<* 0.05, Figure 4A).

There was a similar increase in the expression of phospho-p70^S6K^ at Ser^371^ (140%) and phospho-p70^S6K^ at Thr^389^ (190%) in damaged groups (Cryo and Cryo + Leu) when compared to the non-damaged groups (C and Leu) (*p* *<* 0.05, Figure 4B). The expression of 4E-BP1 and eIF4E (Figure 4B), as well as the amount of ubiquitinated proteins (Figure 4C) were unchanged in all groups analyzed.

### 3.5. Area Density of Connective Tissue and Hydroxyproline Content

**Figure 5:**
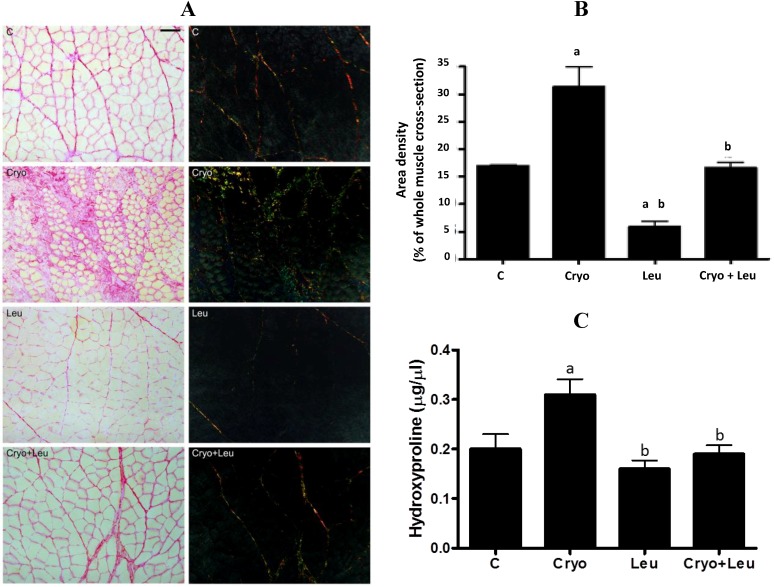
TA muscle cross-sections stained with Sirius red and analyzed under light and polarized microscopes (cross-sections on left and right, respectively, (**A**)). Scale bar = 50 µm. Area density of connective tissue from TA muscles stained with Sirius red (**B**). Hydroxyproline content in muscles (µg/µL; (**C**)). C, control muscles; Cryo, muscles analyzed on Day 10 post-cryolesion; Leu, muscles after 13 days of leucine supplementation; Cryo + Leu, leucine supplemented group analyzed on Day 10 post-cryolesion. Area density of connective tissue is presented as percentage of the whole muscle cross-section. ^a^ *p* < 0.05 *vs.* C; ^b^ *p* < 0.05 *vs.* Cryo. Data are presented as the mean ± SD.

The presence of orange-red collagen fibers in control TA muscle was predominant in the perimysium, and the muscles from the Leu group had a reduction of them (66%, *p* < 0.05, Figure 5A,B) when compared to the C group. On Day 10 post-cryolesion, there was a large amount of green-yellow collagen fibers at both perimysium and endomysium (84%, *p* < 0.05, Figure 5A,B) when compared to the C group. TA muscle from the Cryo + Leu group showed decreased incidence of green-yellow collagen fibers, mainly in the perimysium, when compared to the Cryo group (52%, *p* < 0.05, Figure 5A,B).

Hydroxyproline content in muscles was measured in order to assess the amount of collagen [26]. The hydroxyproline content in muscles from the Leu group was unaltered when compared to those from the C group (Figure 5C). There was an increase of hydroxyproline content in muscles from the Cryo group when compared to those from the C group (55%, *p* *<* 0.05, Figure 5C). Leucine supplementation was able to reduce the hydroxyproline content in muscles from the Cryo + Leu group when compared to those from the Cryo group (39%, *p* *<* 0.05, Figure 5C), reaching values similar to those observed in the C group (Figure 5C).

### 3.6. Activation of TβRI and Smad2/3

**Figure 6:**
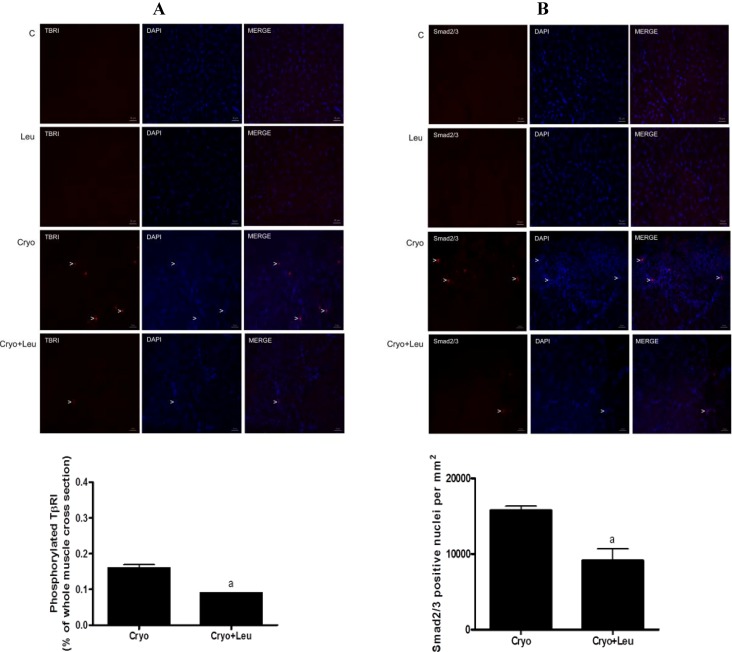
The expression of activated TβRI and Smad2/3 in TA muscles from 10 days post-cryolesion (**A**,**B**), respectively. Activation of TβRI was assessed by the percentage of TβR-I-positive areas. Smad2/3 was assessed by quantifying Smad2/3-positive nuclei in the entire muscle cross-section. C, control muscles; Cryo, damaged muscle analyzed on post-cryolesion Day 10; Leu, muscles after 13 days of leucine supplementation; Cryo + Leu, leucine-supplemented damaged muscle analyzed on post-cryolesion Day 10. Arrowheads indicate phosphorylated TβRI, Smad2/3-positive nucleus, DAPI (nucleus staining), the merge of TβRI and DAPI and the merge of Smad2/3 and DAPI. Scale bar = 50 µm. Results are presented as the mean ± SD. ^a^ *p* < 0.05 *vs.* the Cryo group.

The expression of phosphorylated TβRI was absent in muscles from the C and Leu groups (Figure 6A). There was a decline in the expression of phosphorylated TβRI in TA muscles from the Cryo + Leu group when compared to that from the Cryo group (43%, *p* < 0.05, Figure 6A). There was an absence of Smad2/3-positive nuclei in muscles from the C and Leu groups (Figure 6B). On Day 10 post-cryolesion, TA muscles from the Cryo + Leu group also showed a decrease in the number of Smad2/3-positive nuclei when compared to those from the Cryo group (42%, *p* < 0.05, Figure 6B).

## 4. Discussion

In the present study, we aimed to gain further insight into the effect of leucine supplementation on connective tissue recovery during the skeletal muscle regenerative process after cryolesion, by analyzing the TA muscle, which is representative of fast twitch muscles. Cryolesion is a recognized model that induces necrosis and, subsequently, regeneration, in a well-delineated region of skeletal muscles. Thus, this procedure provides the opportunity to assess the response of skeletal muscle against injury and its regenerative capacity [35,36]. Overall, our results show that leucine supplementation contributes to a better recovery of connective tissue and the consequent function of regenerating TA through attenuation of TGF-β receptor 1 and Smad2/3 activation.

Leucine supplementation alone did not cause a significant increase in the size of myofibers from uninjured TA, which may result from an unaltered rate of protein synthesis and the consequent unchanged expression of MyHC-II observed in our study. These results are in line with the finding that the combination of leucine and glucose supplementation significantly increases tyrosine incorporation into proteins *in vitro* preferentially in fasted slow twitch muscles, such as the diaphragm and soleus, and no effect is detectable in the fasted fast twitch muscle, gastrocnemius [37]. The greater effect of leucine on slow twitch muscle may be related to its more efficient metabolization than in fast twitch muscles [37].

Although in acute conditions of leucine supplementation, such as after a single dose, an increase in the expression of elements from PI3K/Akt/mTOR signaling pathway in uninjured muscle was observed [20,21,22], here and in our previous study [10], no changes in the expression of elements from this pathway were noticed in uninjured muscles after 13 days of leucine supplementation. These effects suggest that the acute activation of PI3K/Akt/mTOR signaling elements promoted by leucine were able to stimulate protein synthesis and to keep it elevated after 13 days, especially in a physiological condition that requires an elevated rate of protein synthesis in order to replace the previously damaged myofibrillar proteins, *i.e.*, after cryolesion. In addition, our results regarding the expression of ubiquitinated proteins suggest that the regaining of myofibrillar proteins in TA muscle at 10 days post-injury may not be mediated by the ubiquitination process.

The increased rate of protein synthesis stimulated by leucine in regenerating muscles may contribute to the elevation of MyHC-II expression in theses muscles at the same time that there was a decrease in neonatal MyHC expression, which suggests that there was an accelerated shift from the neonatal MyHC to adult MyHC-II isoform promoted by leucine in regenerating muscles on post-cryolesion Day 10. However, the increased rate of protein synthesis and MyHC-II expression was reflected only in a slight trend of increase in the caliber of regenerating myofibers upon leucine supplementation in comparison to those from the Cryo group, suggesting that a significant increase in the myofiber size upon leucine supplementation may be detected in periods longer than 10 days post-injury.

Our previous study showed that although leucine supplementation triggered an increase in myofiber size from uninjured soleus muscles, it was not enough to induce an increase in soleus tetanic strength [10]. Interestingly, in the present study, leucine supplementation had a stimulatory effect on contractile function in regenerating TA muscles, such that the decrease in pre-fatigue maximum tetanic strength was avoided in TA muscles, but this effect was not sustained during the fatigue protocol. Accordingly, Hao and coworkers [38] showed that the supplementation of beta-hydroxy-beta-methyl butyrate (HMB), a leucine metabolite, helped to prevent a reduction in maximum tetanic strength in the plantar flexor muscles (fast twitch myofibers) following reloading after atrophy, when compared to the control group. This protection of strength in regenerating TA supplemented with leucine may be also related to the accelerated conversion of neonatal MyHC to adult MyHC that occurs around 10 days post-injury [39,40], as a consequence of increased protein synthesis, which is a typical event that characterize this regenerating period [5]. In fact, Haegens and co-workers [41] showed a myofibrillar protein accretion in cultured skeletal muscle induced by leucine. It is interesting to observe that in the intact TA muscles supplemented with leucine, the pre-fatigue maximum tetanic strength was not increased, possibly because leucine was not able to alter the rate of protein synthesis in these muscles.

The fact that the rate of protein synthesis was unchanged in muscles supplemented with leucine may be related to the effect of performing a single daily dose, a procedure that was chosen to avoid stress caused by excessive animal handling. Recent studies have shown that lower doses of leucine combined with meals applied three-times daily are able to promote a more beneficial effect, demonstrated by the increase in muscle protein synthesis through activation of the PI3K/AKT/mTOR pathway [42,43]. Therefore, futures studies should investigate the potential use of lower doses of leucine applied daily as a therapeutic strategy to accelerate muscle repair.

In order to better understand the effects of leucine in the structure and function of regenerating TA muscles on post-cryolesion Day 10, we also hypothesized that leucine might increase muscle strength by remodelling the ECM. Connective tissue in skeletal muscle is responsible for the transfer of strength from myofibers to the tendons through the fascia and, subsequently, to the bone [44]. Accordingly, Kaasik and coworkers [45] showed that alterations in ECM structure in skeletal muscles were related to changes in muscle strength and motor activity, which led us to determine the area density of intramuscular connective tissue and the amount of collagen in muscles from all groups.

There is a growing body of evidence indicating that ECM can affect muscle function and plasticity, as well as the biological reservoir of muscle stem cells [12]. On Day 10 post-cryolesion, there was a large amount of green-yellow collagen fibers at both perimysium and endomysium of TA muscles. Considering that procollagens and thin collagen fibers appear green and thick collagen fibers appear orange-colored [46,47], it is likely that regenerating muscles at 10 days post-cryolesion have an increased incidence of newly formed green-yellow collagen fibers, which can be corroborated by the increased amount of collagen observed in these muscles. Leucine supplementation significantly decreased the incidence of orange-red collagen fibers in control muscles. However the amount of collagen in these muscles was unaltered, suggesting that leucine supplementation should not represent a health risk for muscles and other organs. In addition, there was a reduction of green-yellow collagen fibers and in the collagen content in cryolesioned muscles supplemented with leucine; thus, this amino acid may contribute to improvement in contractile function of regenerating muscles at 10 days post-injury.

In order to address possible mechanisms involved in the effect of leucine in reducing the amount of collagen in regenerating muscles analyzed at 10 days post-cryolesion, we assessed the activation of the TGF-β/Smad signaling elements, TβR-I and Smad2/3. These signaling elements are well-known mediators of ECM production, being involved in the activation of fibrosis in several tissues, including the skeletal muscle [13,48]. Leucine supplementation significantly reduced the phosphorylation of TβR-I and the number of Smad2/3-positive nuclei in cryolesioned muscles, which corroborates the hypothesis that leucine supplementation minimizes the accumulation of ECM components in regenerating muscles on post-cryolesion Day 10 through attenuation of TGF-β signaling stimulation. These data are in line with the finding that the proteoglycan decorin has a leucine-rich internal region, which modulates the transforming growth factor (TGF)-β-dependent signaling by interacting with the low-density lipoprotein receptor-related protein-1 and subsequently abrogates TGF-β-dependent muscle fibrosis [18].

## 5. Conclusions

In summary, the present study showed that leucine supplementation improves the recovery of structure and function of regenerating TA muscles analyzed on post-injury Day 10. Although leucine supplementation was not able to improve the recovery of regenerating myofiber size, TA muscles presented improvement in contractile performance. Our results suggest that this effect may be related to its capacity to accelerate the shift from neonatal MyHC to adult MyHC, reducing the activation of elements from TGF-β/Smad signaling and, consequently, the amount of collagen, events that characterize this time point. Therefore, future studies should further investigate the possible therapeutic effects of leucine on the modulation of muscle fibrosis in several muscle disorders.
